# The bone marrow microenvironment enhances multiple myeloma progression by exosome-mediated activation of myeloid-derived suppressor cells

**DOI:** 10.18632/oncotarget.6083

**Published:** 2015-11-02

**Authors:** Jinheng Wang, Kim De Veirman, Nathan De Beule, Ken Maes, Elke De Bruyne, Els Van Valckenborgh, Karin Vanderkerken, Eline Menu

**Affiliations:** ^1^ Department of Hematology and Immunology, Myeloma Center Brussels, Vrije Universiteit Brussels (VUB), Brussels, Belgium

**Keywords:** multiple myeloma, bone marrow stromal cells, exosomes, myeloid-derived suppressor cells, immunosuppression

## Abstract

Exosomes, extracellular nanovesicles secreted by various cell types, modulate the bone marrow (BM) microenvironment by regulating angiogenesis, cytokine release, immune response, inflammation, and metastasis. Interactions between bone marrow stromal cells (BMSCs) and multiple myeloma (MM) cells play crucial roles in MM development. We previously reported that BMSC-derived exosomes directly promote MM cell growth, whereas the other possible mechanisms for supporting MM progression by these exosomes are still not clear. Here, we investigated the effect of BMSC-derived exosomes on the MM BM cells with specific emphasis on myeloid-derived suppressor cells (MDSCs). BMSC-derived exosomes were able to be taken up by MM MDSCs and induced their expansion *in vitro*. Moreover, these exosomes directly induced the survival of MDSCs through activating STAT3 and STAT1 pathways and increasing the anti-apoptotic proteins Bcl-xL and Mcl-1. Inhibition of these pathways blocked the enhancement of MDSC survival. Furthermore, these exosomes increased the nitric oxide release from MM MDSCs and enhanced their suppressive activity on T cells. Taken together, our results demonstrate that BMSC-derived exosomes activate MDSCs in the BM through STAT3 and STAT1 pathways, leading to increased immunosuppression which favors MM progression.

## INTRODUCTION

Exosomes are nanometric membrane vesicles derived from late endosomes, released by normal and tumor cells. Exosomes mainly function as mediators of local and systemic communication by transferring mRNAs, microRNAs, and proteins [1]. Uptake of exosomes by recipient cells is mainly carried out through direct fusion with the plasma membrane or endocytic pathways [2]. The exosome, as a communicator, can educate the bone marrow (BM) microenvironment by targeting various cell types in the BM, including macrophages [3], dendritic cells [3], B cells [4, 5], T cells [4, 6], mesenchymal stem cells (MSCs) [7], BM stromal cells (BMSCs) [8], myeloid-derived suppressor cells (MDSCs) [9, 10], as well as tumor cells [8, 11]. Compelling studies reported that exosomes secreted by BM-derived cells or tumor cells maintain and regulate the microenvironment by affecting angiogenesis [12–14], cytokine secretion [8], cell differentiation [15], immune response [1, 16], inflammation [17], and metastasis [18] in the BM.

Multiple myeloma (MM) is a plasma cell malignancy which is predominantly localized in the BM. MM progression largely relies on support from the BM microenvironment, which is mainly composed of stromal cells, endothelial cells, immune cells, and extracellular matrix [19]. It is well studied that stromal cells directly facilitate the MM progression and drug resistance through cell-to-cell contact and cytokine stimulation [20]. In addition, BM-MSCs or BMSCs interact with dendritic cells [21], NK cells [22, 23], and MDSCs [24], to modulate the BM microenvironment, which may indirectly favor tumor growth. We previously reported that exosomes derived from BMSCs could mediate the communication between stromal cells and MM cells and promote MM progression [11]. However, indirect approaches for promoting MM growth through regulation of the BM microenvironment by BMSC-derived exosomes (BMSC exosomes) have not been studied.

MDSCs are immature myeloid cells that are negative regulators of the immune response and which accumulate in secondary lymphoid tissue and in the tumor microenvironment during tumor development [25, 26]. Activated MDSCs promote tumor growth and invasion, immunosuppression, and host immune evasion by suppressing lymphocyte activation and antigen recognition [27]. MDSC expansion and activation are mainly mediated by growth factors secreted by tumor cells, tumor stromal cells, activated T cells and macrophages, and pathogen-infected cells [28]. Exosomes released by cancer cells are also involved in the activation and expansion of MDSC, leading to enhanced immunosuppression which contributes to tumor development [10, 29]. Nevertheless, very little is known about MDSC activation mediated by exosomes derived from other cells in the BM microenvironment.

Immunosuppressive MDSCs can be observed in MM patients and mouse models [30–32]. Our previous studies showed that MDSCs from 5T33MM mice have a higher suppressive capacity than those from naive mice [31]. In the BM microenvironment, stromal cells have been shown to contribute to the expansion and activation of MDSCs through secreting hepatocyte growth factor (HGF) and activating the STAT3 pathway [24]. However, in the MM BM microenvironment, the effects of exosomes released from stromal cells on MDSC activation are still unknown. In the present study, we explored the roles of BMSC exosomes in the expansion and activation of MDSC using the murine 5T33MM model.

## RESULTS

### Uptake of BMSC exosomes by 5T33MM BM cells

**Figure 1:**
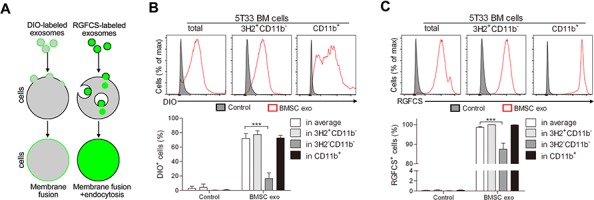
Mechanisms of uptake of BMSC exosomes by MM BM cells **A.** Diagram illustrating how DIO- or RGFCS-labeled exosomes are taken up by cells. BM cells obtained from diseased 5T33MM mice (*n* = 3) were cultured with **B.** DIO- or **C.** RGFCS-labeled BMSC exosomes (100 μg/ml) in 5% serum medium for 24 hours. The uptake of exosomes by MM (3H2^+^CD11b^−^) cells or CD11b^+^ cells or 3H2^−^CD11b^−^ cells was determined by flow cytometry after anti-3H2-APC and anti-CD11b-PE-Cy7 staining. DIO or RGFCS positive cells were regarded as the cells that have already taken up exosomes. *** = *p* < 0.001.

We and others have previously shown that exosomes can mediate the communication between BMSCs and MM cells and the uptake of exosomes by recipient cells is mainly observed through membrane labeling [11, 33]. However, the MM BM microenvironment contains, besides MM cells, various other cell types and here we wished to determine whether BMSC exosomes can be taken up by all these cells. First, to confirm uptake, we labeled the membrane or content of BMSC exosomes using membrane tracker DIO or cell-permeant nucleic acid stains RGFCS respectively and cultured them with 5T33MMvt cells for different time points (Supplementary Figure S1A–S1D). Gradual increase over time of DIO^+^ and RGFCS^+^ cells, as well as enhanced fluorescent signal, were detected after co-culture, suggesting two different ways to take up exosomes by recipient cells, namely membrane fusion and endocytosis (Figure 1A). Next, whole 5T33MM BM was cultured with DIO- or RGFCS-labeled BMSC exosomes and the uptake of exosomes by different subpopulations in the BM cells was determined. Uptake of DIO-labeled exosomes was detected on average in 70% of the BM cells and almost all the cells took up RGFCS-labeled exosomes after 24 hours (Figure 1B and 1C). MM (3H2^+^CD11b^−^) cells and CD11b^+^ cells, which mainly are MDSCs, took up more DIO- or RGFCS-labeled exosomes than the rest of the BM cells (3H2^−^CD11b^−^) (Figure 1B and 1C). Moreover, the mean fluorescence intensity of DIO or RGFCS in CD11b^+^ cells was significantly higher than in the other subpopulations, as well as in total BM cells (Supplementary Figure S1E and S1F), suggesting a greater capacity for taking up exosomes. These results indicate that BMSC exosomes are not only taken up by MM cells, but also by the other BM cells such as MDSCs.

### BMSC exosomes promote the survival of the whole 5T33MM BM cells *in vitro*

**Figure 2:**
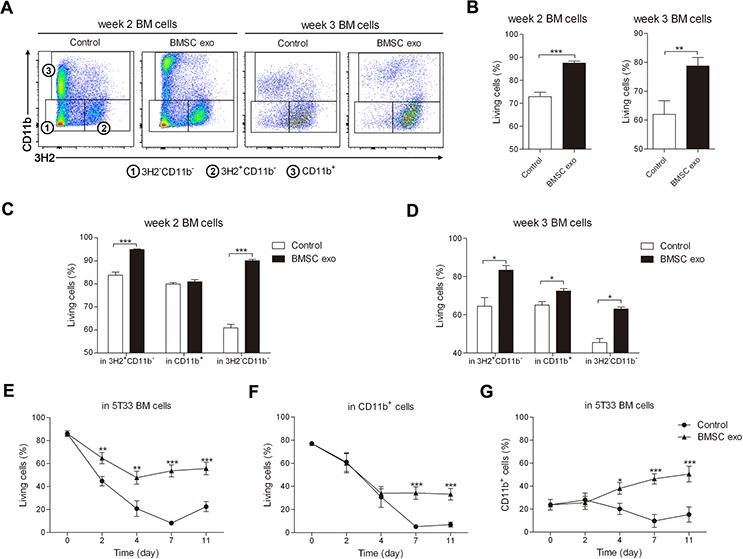
BMSC exosomes promote the survival of BM cells obtained from 5T33MM mice in intermediate and late stage of MM BM cells isolated from mice inoculated with 5T33MM cells for 2 weeks (week 2 BM cells, *n* = 4) or 3 weeks (week 3 BM cells, *n* = 3) were cultured with BMSC exosomes (BMSC exo, 100 μg/ml) in medium with 5% serum for 24 hours and then the cells were stained with anti-3H2-APC, anti-CD11b-PE-Cy7, and Annexin V-FITC. **A.** The gating of the subpopulations 3H2^−^CD11b^−^, 3H2^+^CD11b^−^, and CD11b^+^ cells in week 2 or week 3 5T33MM BM cells after co-culturing with exosomes is shown. **B–D.** The percentage of living (Annexin V^−^) cells after culturing with or without BMSC exosomes was analyzed in: week 2 or week 3 BM population (B); MM (3H2^+^CD11b^−^), CD11b^+^ cells, and 3H2^−^CD11b^−^ in week 2 (C) or week 3 BM cells (D). **E–G.** BM cells isolated from week 3 5T33MM mice (*n* = 3) were cultured with BMSC exosomes (BMSC exo, 50 μg/ml) in 5% serum medium for 11 days. BMSC exosomes were added 3 times a week and the medium was refreshed every week. At different time points, the cells were stained with anti-3H2-APC, anti-CD11b-PE-Cy7, and Annexin V-FITC. (E) The change of living cells in whole BM cells at indicated time points was measured. (F) The change of living cells in CD11b^+^ population and (G) the percentage of CD11b^+^ cells were also evaluated at different time points by flow cytometry. * = *p* < 0.05, ** = *p* < 0.01, *** = *p* < 0.001.

Since BMSC exosomes can be taken up by all the BM cells, we next determined the effect of these exosomes on the different cells. BM cells were isolated from mice 2 weeks (intermediate stage of MM) or 3 weeks (late stage of MM) after inoculation with 5T33MM cells and cultured with BMSC exosomes. After 24 hours of co-culture, subpopulations in the BM cells were altered (Figure 2A) and the survival of the BM cells obtained from week 2 or week 3 5T33MM mice was significantly enhanced (Figure 2B). The number of living MM (3H2^+^CD11b^−^) and 3H2^−^CD11b^−^ cells was significantly higher in both week 2 and week 3 5T33MM BM (Figure 2C and 2D), whereas the enhanced survival of CD11b^+^ cells was mostly observed in week 3 5T33MM BM (Figure 2D). Moreover, the mean fluorescence intensity of CD11b in CD11b^+^ cells was significantly increased by BMSC exosomes (Supplementary Figure S2A). Long-term cultures (11 days) demonstrated that BMSC exosomes could promote the survival of the total BM (Figure 2E) in which the viability of the MM cells was also augmented (Supplementary Figure S2B). Moreover, the survival and percentage of CD11b^+^ cells were significantly increased after respectively 4 and 7 days of co-culture (Figure 2F and 2G). Finally, the presence of CD11b on CD11b^+^ cells was gradually increased by exosomes until day 7 (Supplementary Figure S2C). These data suggest that BMSC exosomes not only affect MM cells, but also MDSCs.

### BMSC exosomes directly promote the survival of MDSC

**Figure 3:**
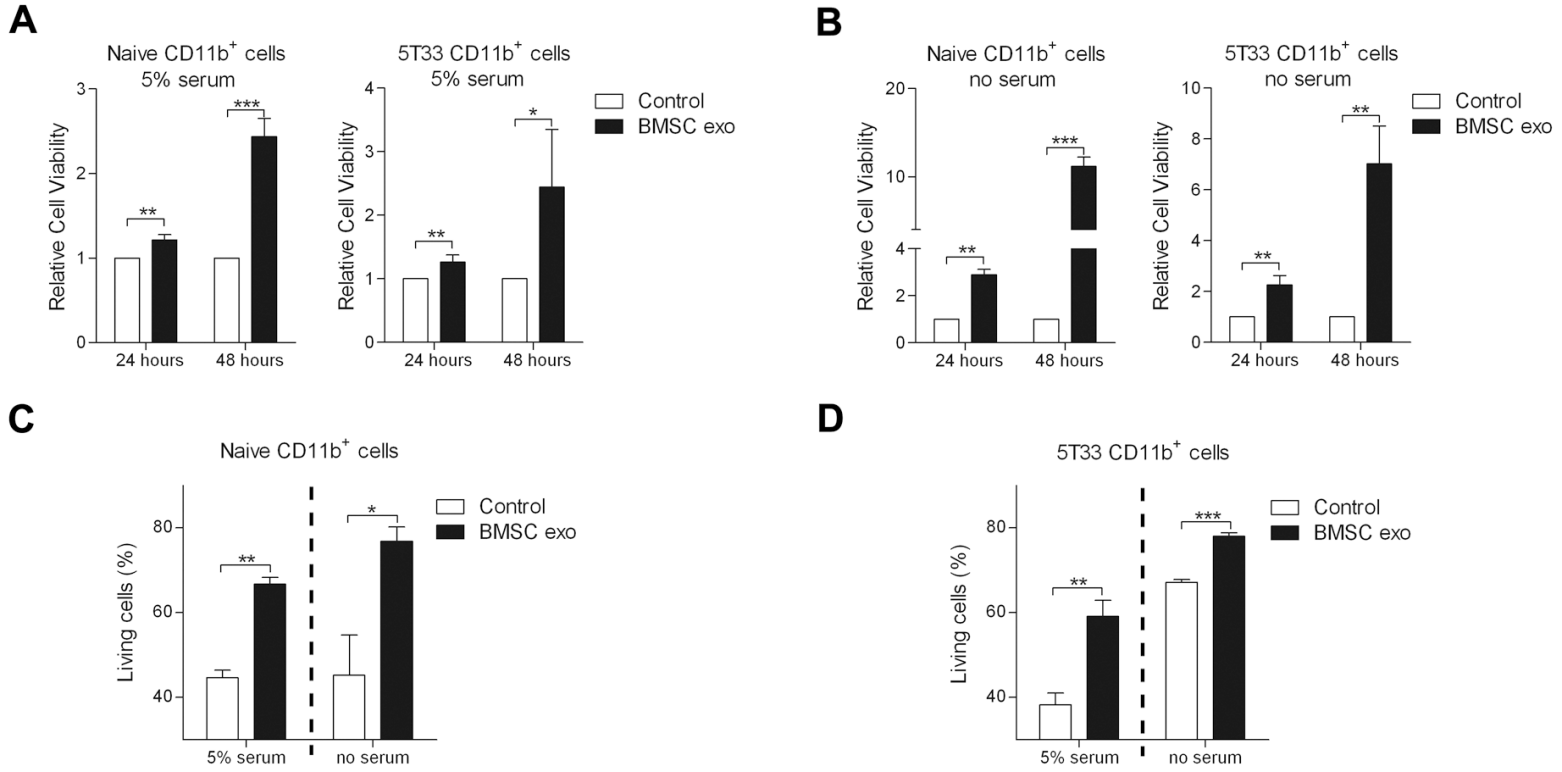
BMSC exosomes induce CD11b+ cell survival CD11b^+^ cells isolated from naive BM (naive CD11b^+^ cells, *n* = 3) or 5T33MM BM (5T33 CD11b^+^ cells, *n* = 3) were cultured with BMSC exosomes (BMSC exo, 100 μg/ml) in **A.** 5% serum or **B.** serum free (no serum) conditions for 24 or 48 hours, and the cell viability was determined by a luminescent viability assay. **C.** Naive (*n* = 3) or **D.** 5T33 CD11b^+^ cells (*n* = 3) were cultured with BMSC exosomes (100 μg/ml) in 5% serum medium for 48 hours or in serum free condition for 24 hours, and the living (Annexin V^−^) cells were determined by flow cytometry. * = *p* < 0.05, ** = *p* < 0.01, *** = *p* < 0.001.

To investigate direct effect of the BMSC exosomes on the MDSC, CD11b^+^ cells were sorted from the BM of naive or 5T33MM mice by MACS and the purity was confirmed (Supplementary Figure S3). 5% serum or serum free conditions were used to determine if BMSC exosomes could affect MDSCs in the presence or absence of growth factors. BMSC exosomes significantly increased the cell viability of naive and 5T33 CD11b^+^ cells in 5% serum medium as measured by a luminescent viability assay (Figure 3A). Moreover, in serum free conditions, BMSC exosomes increased the cell viability of naive and 5T33 CD11b^+^ cells up to 10 times and 7 times respectively (Figure 3B). These data indicate that BMSC exosomes promote MDSC expansion even in the presence of other growth factors. To determine whether this enhanced viability was a result of reduced apoptosis, the percentage of living (Annexin V^−^) naive and 5T33 CD11b^+^ cells was measured by flow cytometry. Figure 3C and 3D demonstrate that exosomes increase the percentage of living cells up to 2 fold in 5% serum or serum free conditions, indicating that reduced apoptosis is not the sole mediator of MDSC expansion.

### BMSC exosomes mainly promote the survival of Ly6G^low^Ly6C^+^ MDSCs

**Figure 4:**
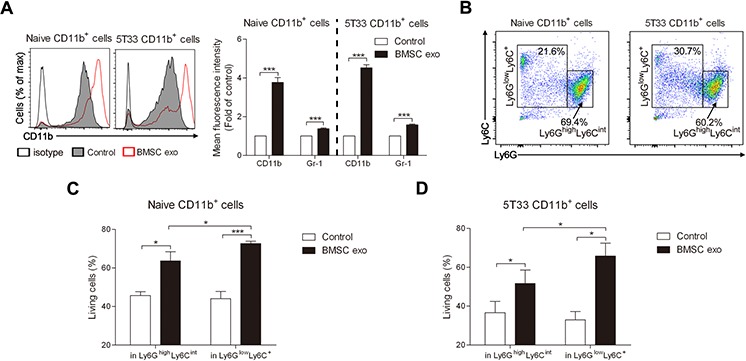
BMSC exosomes mainly induce the survival of CD11b^+^ Ly6G^low^Ly6C^+^ cells **A.** Naive (*n* = 3) or 5T33 CD11b^+^ cells (*n* = 3) were cultured with BMSC exosomes (BMSC exo, 100 μg/ml) in 5% serum medium for 48 hours and then stained with anti-CD11b-PE-Cy7 and anti-Gr-1-APC. Mean fluorescence intensities of CD11b and Gr-1 were measured by flow cytometry. **B.** Subpopulations of naive or 5T33 CD11b^+^ cells were determined using anti-Ly6G-PE-Cy7 and anti-Ly6C-APC staining. **C.** Naive (*n* = 3) or **D.** 5T33 CD11b^+^ cells (*n* = 3) were treated with BMSC exosomes (100 μg/ml) in 5% serum medium for 48 hours and then stained with anti-Ly6G-PE-Cy7, anti-Ly6C-APC, and Annexin V-FITC. The percentages of living (Annexin-V^−^) cells in Ly6G^low^Ly6C^+^ and Ly6G^high^Ly6C^int^ subsets were determined by flow cytometry. * = *p* < 0.05, *** = *p* < 0.001.

Previously, MDSCs have been identified as CD11b^+^Gr-1^+^ cells in mice and two subtypes of MDSC, namely granulocytic MDSCs expressing CD11b^+^Ly6G^high^Ly6C^int^ and monocytic MDSC expressing CD11b^+^Ly6G^low^Ly6C^+^, have been characterized, these subpopulation have distinct phenotypes, morphology, and immunosuppressive mechanisms [31, 34]. As BMSC exosomes directly promote MDSC survival, we next determined the effect of these exosomes on the MDSC subpopulations. After culture with BMSC exosomes, MACS-sorted naive and 5T33 CD11b^+^ cells had 4 times more CD11b expression on their membrane and Gr-1 expression was also slightly increased (Figure 4A), whereas the percentage of CD11b^+^Gr-1^+^ cells was not changed (Supplementary Figure S4A and S4B). An average of 60% CD11b^+^ cells expressed a very high level of CD11b (CD11b^++^) after culture with BMSC exosomes while only 10% CD11b^++^ cells were detected in the control (Supplementary Figure S4C). We next determined the composition of the Ly6G^high^Ly6C^int^ and Ly6G^low^Ly6C^+^ subpopulations in naive and tumor bearing mice and found a clear difference with MM mice having more CD11b^+^Ly6G^low^Ly6C^+^ cells (Figure 4B). This is in line with our previous publication [31]. To determine whether BMSC exosomes are involved in this process, we co-cultured them with naive or 5T33 MDSCs. BMSC exosomes could enhance the survival of both subpopulations and more survival of Ly6G^low^Ly6C^+^ MDSCs, which is composed of inflammatory or classical monocytes, immature myeloid cells, and eosinophils [31], was observed when compared with granulocytic MDSCs (Ly6G^high^Ly6C^int^) (Figure 4C and 4D). These results demonstrate that BMSC exosomes promote the survival of all MDSCs but have a stronger impact on Ly6G^low^Ly6C^+^ MDSCs.

### BMSC exosomes activate STAT1 and STAT3 pathways in MDSCs

**Figure 5:**
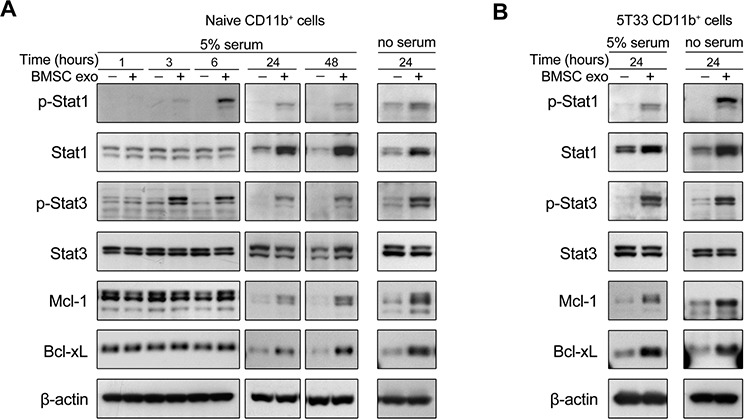
BMSC exosomes activate STAT1 and STAT3 pathways and increase Bcl-xL and Mcl-1 in CD11b^+^ cells **A.** Naive CD11b^+^ cells were cultured with BMSC exosomes (BMSC exo, 100 μg/ml) in 5% serum or serum free (no serum) condition for indicated hours. The total and phosphorylated Stat1 and Stat3, as well as the expression of Bcl-xL and Mcl-1 were detected using western blot. The analysis of β-actin protein was included as a loading control. **B.** 5T33 CD11b^+^ cells were cultured with BMSC exosomes (100 μg/ml) in 5% serum or serum free condition for 24 hours. The total and phosphorylated Stat1 and Stat3, as well as Bcl-xL and Mcl-1 were detected using western blot.

Signal transducer and activator of transcription family proteins (STATs), including Stat1, Stat3, and Stat6, are the main regulators of MDSC expansion and activation and activated Stat3 also contributes to cell survival [28]. Since BMSC exosomes promote MDSC survival, activation of Stat1 and Stat3 in these cells was determined next. BMSC exosomes clearly increased the phosphorylation of Stat1 and Stat3 (p-Stat1 and p-Stat3) in naive and 5T33 CD11b^+^ cells in both 5% serum and serum free conditions (Figure 5A and 5B). Elevated protein level of Stat1 was observed in CD11b^+^ cells cultured with the exosomes (Figure 5A and 5B). Two anti-apoptotic proteins Mcl-1 and Bcl-xL, mainly regulated by the STAT3 pathway, were also clearly increased by the exosomes after 24 hours (Figure 5A and 5B).

### Inhibition of STAT1 and STAT3 pathways reduce BMSC exosome effects on MDSC survival

**Figure 6:**
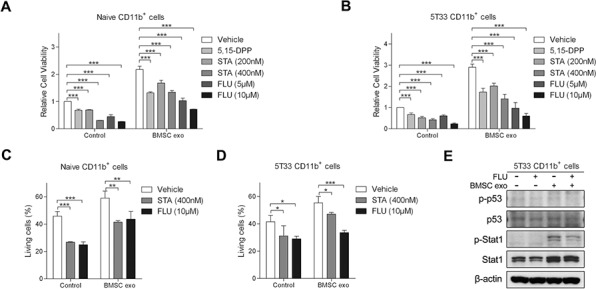
Inhibitors of STAT1 and STAT3 pathways suppress BMSC exosomes-induced survival of CD11b^+^ cells **A.** Naive (*n* = 3) or **B.** 5T33 CD11b^+^ cells (*n* = 3) in 5% serum medium were cultured with or without BMSC exosomes (100 μg/ml) in the absence or presence of 5, 15-DPP, stattic (STA), or fludarabine (FLU) for 48 hours and the cell viability was determined by a luminescent viability assay. **C.** Naive (*n* = 3) or **D.** 5T33 CD11b^+^ cells (*n* = 3) in 5% serum medium were cultured with or without BMSC exosomes in the absence or presence of STA or FLU for 48 hours and living (Annexin V^−^) cells were determined by flow cytometry after Annexin V-FITC staining. **E.** 5T33 CD11b^+^ cells in 5% serum medium were cultured with or without BMSC exosomes in the absence or presence of FLU (10 μM) for 24 hours and phosphorylation of p53 and Stat1 (p-p53 and p-Stat1), as well as p53 and Stat1 were detected by western blot. β-actin protein was included as a loading control. * = *p* < 0.05, ** = *p* < 0.01, *** = *p* < 0.001.

Several papers have suggested how MDSCs can be activated by tumor cells. Granulocyte/macrophage colony-stimulating factor (GM-CSF) is a soluble factor involved in MDSC activation and our group demonstrated that a blocking antibody of GM-CSF can abrogate MM induced pro-survival effects on MDSCs [25]. Membrane-associated Hsp70 from tumor-derived exosomes has been shown to activate MDSC through STAT3 pathways [29]. Here, we used blocking antibodies to examine the involvement of GM-CSF and Hsp70 in BMSC exosome-induced effects. However, these two blocking antibodies did not affect CD11b^+^ cell viability in the absence or presence of BMSC exosomes, suggesting that exosome-induced activation of MDSC is independent of GM-CSF and membrane-associated Hsp70 (Supplementary Figure S5A and S5B). Inhibitors of STAT1 and STAT3 pathways were next used to confirm the involvement of these two pathways in CD11b^+^ cell survival induced by exosomes. The STAT1 inhibitor fludarabine and two STAT3 inhibitors 5,15-DPP and stattic, inhibited CD11b^+^ cell viability even in the presence of BMSC exosomes (Figure 6A and 6B). They also significantly suppressed BMSC exosome-mediated survival of both naive and 5T33 CD11b^+^ cells (Figure 6C and 6D), confirming the participation of STAT1 and STAT3 pathways in MDSC survival. Fludarabine is a well-used Stat1 inhibitor [35, 36], but it has been shown to affect other pathways such as p53 [37], therefore we examined whether p53 activation was impacted in MDSC. In 5T33 CD11b^+^ cells, fludarabine reduced the phosphorylation of Stat1 in the presence of BMSC exosomes, whereas p53 and its phosphorylation were not changed (Figure 6E).

### BMSC exosomes activate the STAT3 pathway in MDSCs *in vivo* and enhance their capacity for T cell suppression

**Figure 7:**
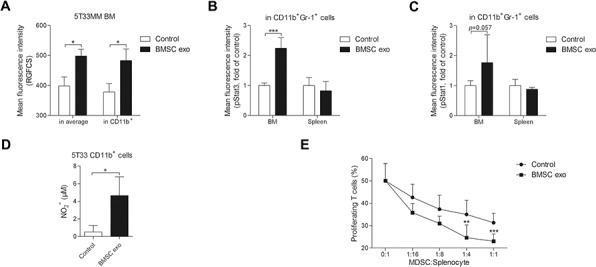
BMSC exosomes activate MDSCs *in vivo* and enhance their capability of T cell suppression **A.** 200 μg RGFCS-labeled BMSC exosomes (BMSC exo) or RGFCS control were intravenously injected into week 2 5T33MM mice (*n* = 3) for 24 hours. The BM cells were isolated and stained with anti-CD11b-PE-Cy7. Mean florescence intensities of RGFCS in the total BM cells (in average) and CD11b^+^ cells were determined by flow cytometry. **B.** and **C.** 200 μg BMSC exosomes or PBS (control) were intravenously injected into week 2 5T33MM mice (*n* = 4) for 24 hours. Thereafter, the bone marrow and spleen cells were isolated and stained with anti-CD11b-PE-Cy7, anti-Gr-1-APC, anti-p-Stat3-Alexa Fluor 488, and anti-p-Stat1-PE. Mean fluorescence intensities of (B) p-Stat3 or (C) p-Stat1 in CD11b^+^Gr-1^+^ cells were measured by flow cytometry. **D.** CD11b^+^ cells isolated from 5T33MM mice (*n* = 3) were cultured with BMSC exosomes for 48 hours and the concentration of NO in supernatant was measured. **E.** Splenocytes obtained from naive mice were labeled with CFSE, stimulated with CD3/CD28 Dynabeads, and cultured with indicated ratio of CD11b^+^ cells obtained from week 2 5T33MM mice (*n* = 8) intravenously injected with PBS (control) or 200 μg BMSC exosomes. After 3 days of culture, the cells were stained with anti-CD3-PE-Cy7 and 7-AAD and the percentage of proliferating cells within gated CD3^+^7-AAD^−^ cells was determined by flow cytometry. * = *p* < 0.05, ** = *p* < 0.01, *** = *p* < 0.001.

To confirm the uptake of exosomes by MDSC *in vivo*, RGFCS-labeled BMSC exosomes were intravenously injected into 5T33MM mice and after 24 hours, an increase of mean fluorescence of RGFCS in the BM and CD11b^+^ cells was observed (Figure 7A). In addition, *in vivo* injection of BMSC exosomes increased activation of Stat3 (p-Stat3) in BM CD11b^+^Gr-1^+^ cells (Figure 7B). p-Stat1 also showed a non significant (*p* = 0.057) trend to increase in BM MDSCs after injection with exosomes (Figure 7C). Activated MDSCs tend to release more nitric oxide (NO) production which contributes to the inhibition of T cells [28]. BMSC exosomes increased the release of NO from 5T33 CD11b^+^ cells (Figure 7D), whereas NO production was undetectable in naive CD11b^+^ cells even in the presence of BMSC exosomes (data not shown). Moreover, MDSCs from 5T33MM mice injected with BMSC exosomes exerted a stronger immunosuppressive effect on T cell proliferation compared to those from 5T33MM mice injected with PBS (Figure 7E).

## DISCUSSION

**Figure 8:**
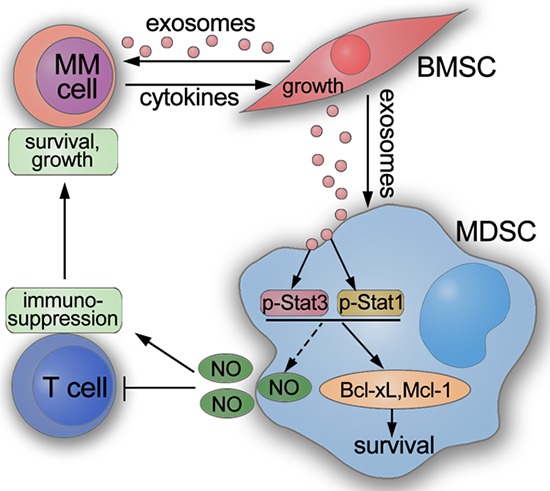
Schematic showing how BMSC exosomes indirectly favor MM cells through activating MDSCs

Activation of MDSC by tumor exosomes has already been investigated in solid tumor models [10, 29], however the influence of exosomes from the surrounding tissue has not been thoroughly examined yet. Since MM develops in the BM, we sought to investigate the effect of BMSC-derived exosomes on MDSC activation. Our results have identified BMSC exosomes as novel mediators for MDSC activation which leads to an enhancement of the immunosuppressive function of MDSC in the MM BM. Through culturing of BMSC exosomes with MM BM cells, we demonstrated that these exosomes can be taken up not only by MM cells but also by MDSCs and that these cells are affected in the short and long term. BMSC exosomes directly promote the survival of MDSCs by an enhanced activation of STAT1 and STAT3 pathways. The activated MM MDSCs in the 5T33MM mouse model acquired an enhanced capacity for T cell suppression which facilitates immune escape of the MM cells. Our work proposes an indirect mechanism for promoting MM progression by BMSC exosomes: exosomes released from BMSCs induce MDSC survival and elevate their immunosuppressive capacity, leading to T cell suppression which favors MM development (Figure 8).

By labeling the membrane or content of BMSC exosomes with DIO or RGFCS, we identified that these exosomes can be taken up by all BM cells. Extracellular vesicles, including exosomes and microvesicles, deliver their contents of proteins, RNAs, and lipids through direct fusing with the cell plasma membrane or being endocytosed and internalized by recipient cells [2]. Here, we detected the uptake of both DIO- and RGFCS- labeled exosomes by MM cells, as well as CD11b^+^ cells, with CD11b^+^ cells having a higher ability for taking up exosomes. These results can be explained by the fact that CD11b^+^ cells mainly are MDSCs (because nearly all of them co-express Gr-1) [31] which have bigger membranes than MM cells or other lymphocytes, leading to an increased opportunity for fusing with exosomes.

It has been previously demonstrated that accumulation of activated MDSCs in the BM is observed at early stages of MM and it has been shown that they play a critical role in MM progression by inhibiting T cell function [30]. However, the mechanisms behind this increase are not completely understood yet. Very few studies have demonstrated the relationship between exosomes and MDSC expansion and they are mainly focused on exosomes secreted from tumor cells which induce immunosuppressive functions in MDSC [10, 29], whereas the effects of exosomes derived from the other cells on MDSC have not been studied. Our finding that BMSC exosomes directly induced survival of MDSC even in the presence of growth factors would emphasize the importance of exosomes derived from non-tumor cells in tumor growth through educating the BM microenvironment. CD11b can regulate leukocyte adhesion and cell migration [34] and BMSC exosomes dramatically increased its expression on the membrane of MDSC which may lead to the enhancement of their ability to migrate to secondary lymphoid tissue. In mice, two major subsets of MDSCs, namely granulocytic MDSCs and monocytic MDSCs, have been identified [34] and they have different functions in cancer and infectious and autoimmune diseases [28, 34, 38, 39]. BMSC exosomes were able to promote the survival of both subsets and specifically induced more survival of CD11b^+^Ly6G^low^Ly6C^+^ cells which consist of inflammatory or classical monocytes, immature myeloid cells, and eosinophils. Our group has reported that CD11b^+^LyG^low^ cells are increased in 5T33MM BM and that they have a higher immunosuppressive capacity than CD11b^+^Ly6G^high^ cells [31]. As a part of the BM microenvironment, BMSCs may contribute to the increase of CD11b^+^LyG^low^ cells and enhancement of immunosuppression through exosome secretion during MM progression.

Most of the factors that induce MDSC activation trigger STAT signaling pathways, including STAT3, STAT6, and STAT1, to induce cell survival, proliferation, differentiation, and expansion of MDSC [28, 40–42]. Exosomes derived from mouse tumor cells could enhance the suppressive activity of MDSCs via the STAT3 pathways [29]. STAT1 is a transcription factor which is involved in the upregulation of immune suppressive factors by MDSCs upon stimulation of IFN-γ. Moreover, Stat1^−/−^ mice are not able to increase these factors and therefore can not inhibit T cell functions [43]. In our work, BMSC exosomes increased the activation of Stat1 and Stat3 in MDSCs from both naive and 5T33MM mice, suggesting a potential role of these exosomes in immunosuppression. In addition, pro-survival proteins Bcl-xL and Mcl-1 were also elevated by BMSC exosomes. By using inhibitors for STAT3 and STAT1 pathways, BMSC exosome-mediated MDSC survival was significantly inhibited, confirming the involvement of these pathways in exosome-induced cell survival. However, since control conditions were also affected by these inhibitors, we cannot rule out the involvement of other pathways. A recent study has shown that MDSC could directly mediate MM cell growth through PD1/PD-L1 signaling [44], therefore increased survival of MDSC through BMSC exosomes may directly affect MM cell growth.

It has been suggested that the expansion and activation of MDSCs is influenced by different factors produced by tumor cells, activated T cells, and tumor stromal cells [28]. Matrix metalloproteinase-9 (MMP-9), MMP-8, and IL-8 produced by stromal cells in solid tumors facilitate MDSC expansion and human bone marrow MSCs also mediate expansion of MDSCs via hepatocyte growth factor (HGF) [24, 45, 46]. Extensive evidence has confirmed GM-CSF as an efficient stimulator for the immunosuppressive activity of MDSC and blocking the GM-CSF receptor blunts MDSC activation and expansion [25, 47, 48]. Neutralization of the binding between Hsp70 on the membrane of exosomes derived from solid tumors and MDSCs also abrogates exosome-induced MDSC activation [29]. However, in our system, increase in MDSC cell viability induced by BMSC exosomes was not influenced by neutralizing antibodies for GM-CSF and Hsp70, implying the lack of involvement of GM-CSF and Hsp-70 in this cross talk, underscoring the difference between the exosomes derived from tumor cell and BMSC. Since BMSCs can promote MM cell growth through secretion of cytokines such as vascular endothelial growth factor, IL-6, and HGF [20], which have also been suggested to be involved in MDSC activation [49], it is possible that these factors play a role in BMSC exosome stimulation.

Activated MDSC suppress T cell function mainly through two mechanisms: direct cell-cell contact and secretion of soluble mediators [28]. Increased NO production is commonly observed after MDSC activation [39, 50] and MDSC induce T cell suppression through various mechanisms that involve the inhibition of MHC class II expression [51] and induction of T cell apoptosis [52]. Here, we show for the first time that BMSC exosomes induced more production of NO from 5T33MM BM MDSC and that they enhanced the T cell suppressive capacity of MDSCs *in vivo*. Our data, together with other studies showing that increased NO production induces suppression of T cell proliferation [28, 53], suggest that BMSC exosomes enhance immunosuppression through promoting NO release in MDSCs. Also, our group has shown that T-cell suppression by Ly6G^low^ MDSCs was partially mediated through NO [31]. Moreover, an increase in the number of MDSCs induced by BMSC exosomes may also contribute to the enhancement of suppression through cell-cell contact.

In conclusion, BMSC exosomes not only directly promote MM survival but also indirectly favor MM progression through enhancing the immunosuppressive activity of MDSC in the BM. Our findings not only address the role of BMSC exosomes in the BM microenvironment but also expand the understanding of the participation of these exosomes in MM progression. Moreover, our results strengthen the usefulness of STAT inhibitors in MM treatment through targeting of MDSC activation.

## MATERIALS AND METHODS

### Mice and 5T33MM model

C57BL/KaLwRij mice used in this study were purchased from Harlan Laboratories (Horst, The Netherlands). The 5T33MM model is propagated by intravenous injection of diseased MM BM cells into 8–10 weeks female naive C57BL/KaLwRij mice. All the mice were housed and treated following conditions approved by the Ethical Committee for Animal Experiments of the Vrije Universiteit Brussel (license no. LA1230281).

### Cell culture, blocking antibodies, and reagents

Primary BMSCs were isolated from diseased 5T33MM mice and maintained in DMEM medium (Lonza, Visp, Switzerland) supplemented with 10% fetal calf serum (HyClone, Logan, UT, USA), 10% horse serum (Invitrogen, Carlsbad, CA, USA), 100 U/mL penicillin/streptomycin and 2 mM L-glutamine (Lonza). BMSCs were used at the second to tenth passage without mycoplasma contamination. Anti-mouse GM-CSF (16–7331) and IgG isotype control (16–4321) were bought from eBioscience (San Diego, CA USA). Anti-Hsp70 (MA3–009) was purchased from Thermo Scientific (Rockford, IL, USA). The lipophilic tracer DIO and SYTO RNASelect green fluorescent cell stain (RGFCS) were purchased from Life Technologies (Carlsbad, CA, USA). 5, 15-DPP was purchased from Sigma (St Louis, MO, USA). Stattic and fludarabine were bought from Selleckchem (Houston, TX, USA).

### Exosome isolation

Exosome were isolated from conditioned medium as previously described [11], but with a small modification. Briefly, BMSCs were cultured without serum for 48 hours and conditioned medium was filtered using 0.22-μm pore filter. Pierce protein concentrator (150KD) (Thermo Scientific) was used to concentrate the filtered medium and to remove the soluble proteins (less than 150kD), as well as smaller particles (less than ∼15nm). Concentrated medium were filtered with 0.22-μm pore filter again and incubated with ExoQuick-TC exosome precipitation solution (System Biosciences, Mountain View, CA, USA) at 4°C overnight. Exosomes were subsequently pelleted by centrifugation at 1,500g for 30 minutes and suspended in PBS or serum-free medium. Protein quantification was performed as previously described [11].

### Fluorescent labeling of exosomes

Exosome suspension was incubated with DIO cell-labeling solution for 30 minutes at 37°C according to the manufacturer's instruction and exosomes were reprecipitated using ExoQuick-TC exosome precipitation solution. DIO-containing medium without exosomes was used as a procedure control. For RGFCS staining, exosome suspension or medium without exosomes (control) were incubated with RGFCS (10 μM) at 37°C for 30 minutes. An Exosome Spin Column (Life Technologies) was used to remove the unincorporated DIO or RGFCS from the labeled exosomes. DIO or RGFCS control solutions were also processed using Exosome Spin Columns and the flow-through was collected and used as a parallel control.

### Flow cytometry

For staining the membrane antigens of BM cells, single-cell suspension was prepared. After treatment with exosomes, 1 × 10^6^ cells were resuspended in PBS with 5% BSA and stained with PE-Cy7-conjugated antibodies to CD11b (anti-CD11b-PE-Cy7, 101216, BioLegend, San Diego, CA, USA) and with 3H2, an anti-idiotype antibody against 5T33MM cells [54], followed by an APC-conjugated rat anti mouse IgG1 (550874, BD Biosciences, San Jose, CA, USA). For staining the membrane markers of MDSCs, the cells were stained with anti-Gr-1-APC (17–5931, eBioscience) and anti-CD11b-PE-Cy7 or with anti-Ly6G-PE-Cy7 (127618, BioLegend) and anti-Ly6C-APC (560595, BD Biosciences). Staining with Annexin V-fluorescein isothiocyanate (Annexin V-FITC, 556419, BD Biosciences) was used to determine the apoptotic cells. For staining intracellular p-Stat3 and p-Stat1 in MDSCs, cells were first fixed with formaldehyde and methanol, followed by staining with anti-Gr-1-APC, anti-CD11b-PE-Cy7, anti-p-Stat3-Alexa Fluor 488 (557814, BD Biosciences), and anti-p-Stat1-PE (612564, BD Biosciences). The percentage of a certain population and mean fluorescence intensity was evaluated using a FACSCanto flow cytometer (BD Biosciences) and Flowjo software (TreeStar, Ashland, OR, USA).

### Magnetic-activated cell sorting (MACS) for CD11b^+^ cells

After removing red blood cells, BM cells obtained from naive or 5T33MM mice were incubated with CD11b Microbeads (Miltenyi Biotec, Bergisch Gladbach, Germany) for 15 minutes at 4°C. CD11b^+^ cells were then separated using LS Columns (Miltenyi Biotec) and MACS separator (Miltenyi Biotec) according to the manufacturer's instruction. The purity of CD11b^+^ cells was determined using flow cytometry after staining with anti-CD11b-PE-Cy7.

### Cell viability and cell apoptosis assays

5 × 10^4^ sorted naive or 5T33MM CD11b^+^ cells were treated with or without BMSC-derived exosomes in the absence or presence of indicated inhibitors for 24 or 48 hours. Then, cell viability was measured by Cell Titer glo Luminescent Viability assay (Promega, Madison, WI, USA) according to the manufacturer's instructions. For the cell apoptosis assay, 5 × 10^5^ CD11b^+^ cells were seeded in a 24-well plate and treated with exosomes and inhibitors. Apoptotic cells were determined using flow cytometry after Annexin V-FITC staining.

### Western blot

Western blot was performed as described previously [11] and antibodies for β-actin (4967), Bcl-xL (2764), Mcl-1 (5453), Stat1 (9172), p-Stat1 (Tyr701) (9167), Stat3 (4904), p-Stat3 (Tyr705) (9138), p-p53 (9281) and p53 (2524) as well as horseradish peroxidase (HRP)-linked anti-mouse (7076) and -rabbit (7074) IgG, purchased from Cell Signaling Technology (Bioké, Leiden, The Netherlands) were used.

### Nitric oxide (NO) measurement

CD11b^+^ cells (3 × 10^6^/ml) isolated from 5T33MM BM were cultured with exosomes for 48 hours and the supernatants were collected for NO measurement. NO was measured as nitrite using a Griess reagent kit (Life Technologies) according to the manufacturer's instruction. Briefly, 130 μl Milli-Q water was mixed with 20 μl of mixture solution containing sulfanilic acid and N-(1-naphthyl) ethylenediamine dihydrochloride, followed by adding 150 μl culture supernatant. After 30 minutes incubation, absorbance was measured at 540nm in a spectrophotometric microplate reader.

### *In vivo* study

Mice were inoculated with 5 × 10^5^ 5T33MM cells and after 2 weeks, they were randomly divided and intravenously injected with 200 μg BMSC exosomes or PBS. After 24 hours, the total BM and spleen cells were isolated and the levels of p-Stat1 and p-Stat3 in CD11b^+^GR-1^+^ cells were measured using flow cytometry. CD11b^+^ cells were sorted from these BM cells using MACS and the immunosuppressive capacity of these cells was determined using T cell proliferation assay.

### T cell proliferation assay

Splenocytes were isolated from naive mice and stained with CFSE after removing red blood cells. CFSE-labeled cells were cultured with RPMI1640 medium supplemented with 10% HEPES (Sigma) and 20 μM β-mercaptoethanol (Sigma) for 20–30 minutes. Then, they were seeded into a 96-well plate (1 × 10^5^ cells/well) and stimulated with CD3/CD28 Dynabeads (Life Technologies). CD11b^+^ cells obtained from week 2 5T33MM mice injected with PBS or BMSC exosomes were added to splenocytes at multiple ratios and cultured for 3 days. All the cells were stained with anti-CD3-PE-Cy7 (100220, BioLegend), 7-amino-actinomycin D (7-AAD, BD Biosciences) and proliferation of living T cells (CD3^+^7-AAD^−^ cells) was determined with CFSE dilution by flow cytometry.

### Statistical analysis

Results were analyzed with Graphpad prism 5 software. Mann-Whitney test or One-way ANOVA was used to determine the statistical significance. Error bars represent mean ± standard deviation (S.D.). *p* < 0.05 was regarded as statistically significant.

## SUPPLEMENTARY FIGURES AND TABLES


