# Supplementary material for: The bone marrow microenvironment enhances multiple myeloma progression by exosome-mediated activation of myeloid-derived suppressor cells
Source: Oncotarget. 2015 Nov 2;6(41):43992–4004. doi: 10.18632/oncotarget.6083 (PMC4791281; doi:10.18632/oncotarget.6083)
Supplement: Supplementary file 1 [file oncotarget-06-43992-s001.pdf]

SUPPLEMENTARY FIGURES

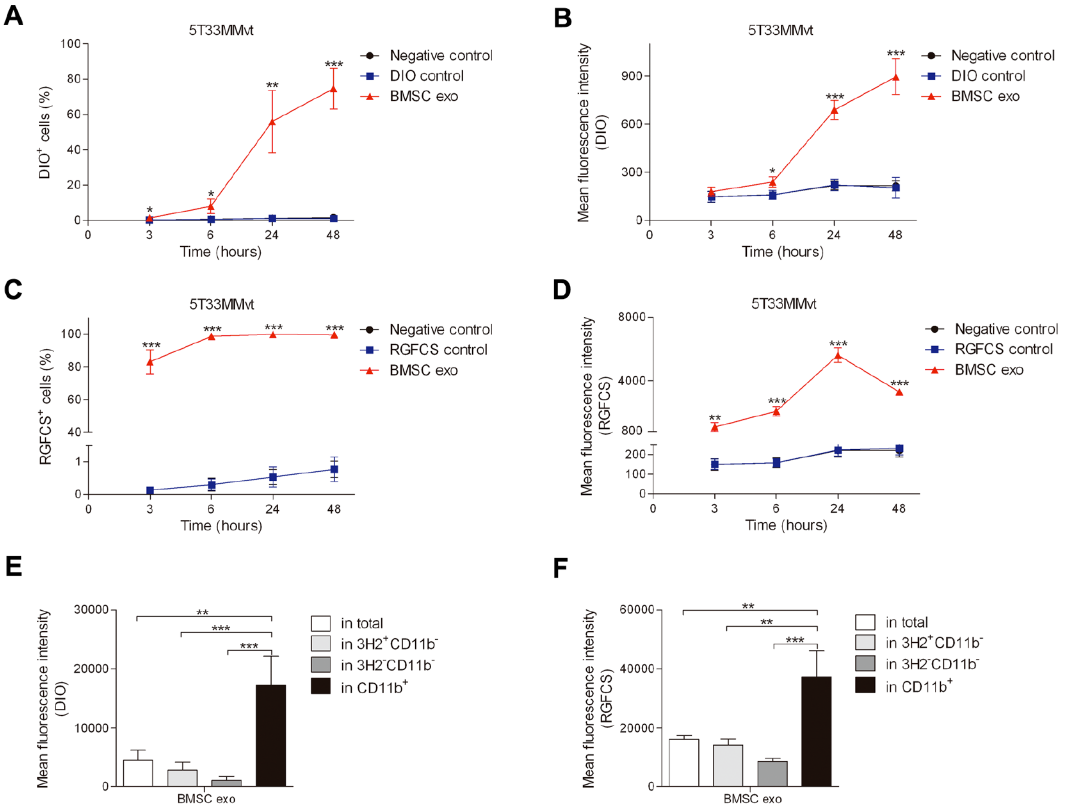

Supplementary Figure S1: Exosome uptake in the different BM populations. 5T33MMvt cells were cultured with DIO- or RGFCS-labeled BMSC-derived exosomes (BMSC exo, *n* = 3, 100 µg/ml) for indicated times. The percentage of **A.** DIO$^+$ or **C.** RGFCS$^+$ cells was determined at different time points. Mean fluorescence intensity of **B.** DIO or **D.** RGFCS in the cells was also evaluated. The BM cells obtained from diseased 5T33MM mice (*n* = 3) were cultured with **E.** DIO- or **F.** RGFCS-labeled BMSC-derived exosomes (100 µg/ml) in 5% serum medium for 24 hours. Mean fluorescence intensity of DIO or RGFCS in the different subpopulations was evaluated by FACS after being stained with anti-3H2-APC and anti-CD11b-PE-Cy7. * = $p < 0.05$, ** = $p < 0.01$, *** = $p < 0.001$.

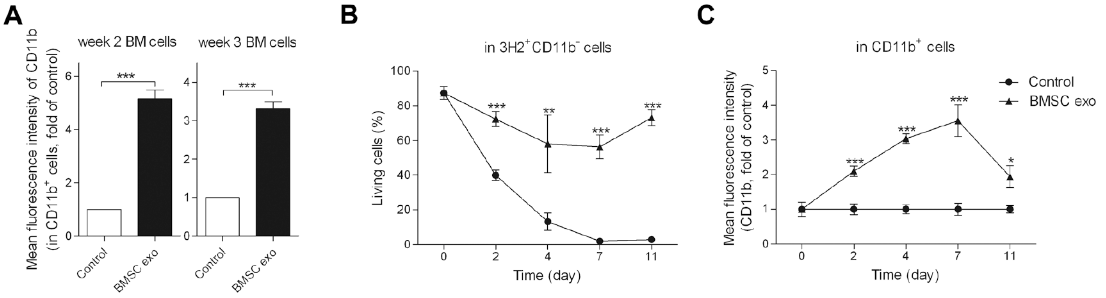

Supplementary Figure S2: Effect of exosomes on BM culture. A. BM cells isolated from week 2 (*n* = 3) or week 3 5T33MM mice (*n* = 3) were cultured with BMSC-derived exosomes (BMSC exo, 100 µg/ml) in medium with 5% serum for 24 hours and the mean fluorescence intensity of CD11b within gated CD11b$^+$ population was measured by flow cytometry. **B.** BM cells isolated from week 3 5T33MM mice (*n* = 3) were cultured with BMSC-derived exosomes (BMSC exo, 50 µg/ml) in medium with 5% serum for 11 days and the percentage of living MM cells, as well as **C.** the mean fluorescence intensity of CD11b within gated CD11b$^+$ population, were measured by flow cytometry. * = $p < 0.05$, ** = $p < 0.01$, *** = $p < 0.001$.

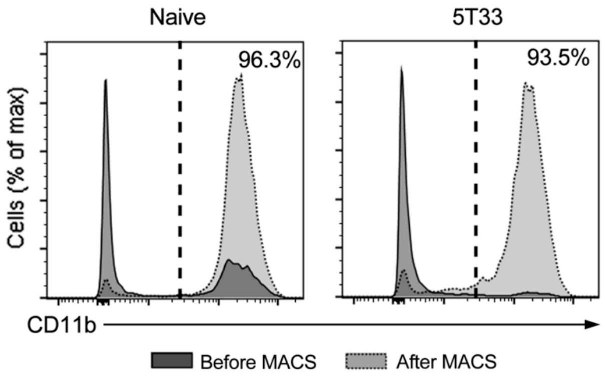

Supplementary Figure S3: Purity of CD11b$^+$ after MACS. CD11b$^+$ cells were sorted from the BM of naive and 5T33MM mice using MACS and the purity was determined using flow cytometry after anti-CD11b-PE-Cy7 staining

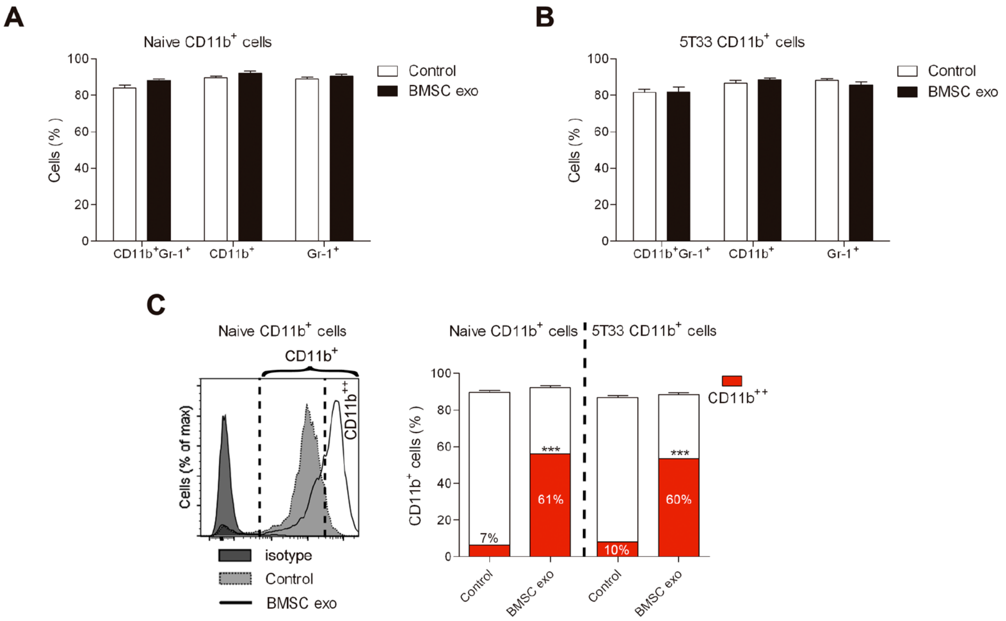

Supplementary Figure S4: BMSC-derived exosomes increased the CD11b^{++} cells. A. Naive and **B.** 5T33 CD11b$^+$ cells (n = 3) were cultured with BMSC-derived exosomes in medium with 5% serum for 48 hours and stained with anti-CD11b-PE-Cy7 and anti-Gr-1-APC. The percentages of CD11b$^+$Gr-1$^+$ cells, CD11b$^+$ cells and Gr-1$^+$ cells were detected by flow cytometry. **C.** The percentage of CD11b^{++} cells within gated CD11b$^+$ cells was measured by flow cytometry after culturing CD11b$^+$ cells with BMSC-derived exosomes. *** = $p < 0.001$.

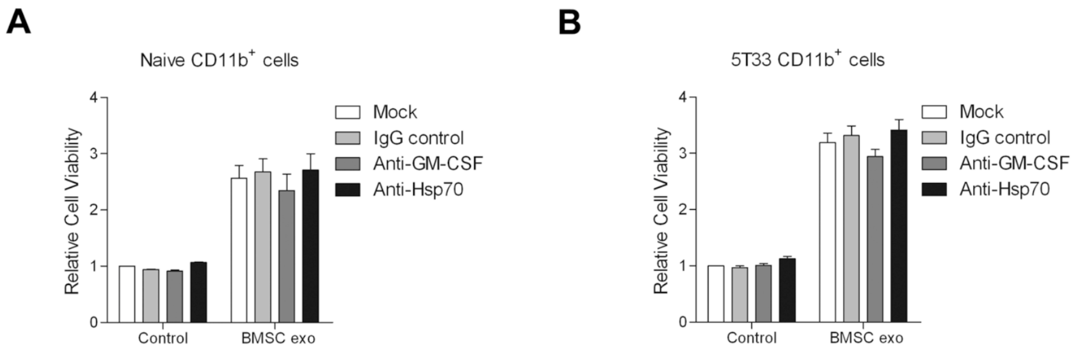

Supplementary Figure S5: Blocking antibodies of GM-CSF and Hsp70 do not affect MDSC viability in the presence of BMSC exosomes. A. Naive ($n = 3$) or **B.** 5T33 CD11b$^+$ cells ($n = 3$) in 5% serum medium were cultured with or without BMSC exosomes (BMSC exo, 100 µg/ml) in the absence or presence of indicated blocking antibodies (anti-GM-CSF, 10 µg/ml; anti-Hsp70, 1µl/ml) for 48 hours and the cell viability was determined by a luminescent viability assay.
